# Verification of the factors of individuality through avatar’s speech generation system

**DOI:** 10.1038/s41598-026-47224-z

**Published:** 2026-06-17

**Authors:** Yuya Komai, Takahisa Uchida, Hiroko Kamide, Hiroshi Ishiguro

**Affiliations:** 1 Osaka University, Osaka, Japan; 2 Kyoto University, Kyoto, Japan

**Keywords:** Cultural and media studies, Cultural and media studies, Psychology, Psychology, Science, technology and society

## Abstract

In recent years, extensive research has been conducted on avatars, and multiple studies have demonstrated their effectiveness as a medium for remote operation. While avatars are effective when teleoperated, they must also be capable of autonomous behavior in the absence of an operator. In particular, avatars whose appearance closely resembles that of a real individual need to possess conversational abilities that reflect the personality of the person being modeled. This paper presents the development of a speech generation system that produces personality-consistent utterances using a large language model (LLM) and speech synthesis technology. We call this system AvatarLLM. Through system evaluation, we examined the factors contributing to the perception of individuality. Experimental results indicated that the utterances generated by AvatarLLM were perceived as more likely reproducing the modeled individual than those of the actual person. Furthermore, we found that the perceived identity of the utterances could influence the perceived identity of the voice itself.

## Introduction

In recent years, extensive research has been conducted on avatars. The term “avatar” refers to a “representation of oneself” in various forms. Kumazaki et al.^1^ demonstrated that a communication training system using teleoperated robots is effective in improving the communication skills of individuals with autism spectrum disorder (ASD). Additionally, Baba et al.^2^ showed that the use of avatars in public service provision can reduce workload while maintaining performance equivalent to face-to-face service.

Avatars can exist as CG agents in virtual spaces or as robots in the real world. By remotely operating avatars, individuals can participate in various social activities. Kato et al.^3^ demonstrated the effectiveness of a remote education system using android robots. Thus, avatars serve as useful media for remote operation. Furthermore, autonomous behavior in the absence of an operator enhances human capabilities by allowing avatars to participate in social activities and build social relationships on behalf of the user.

“When providing dialogue capabilities to avatars that closely resemble real individuals, it is essential that they possess conversational abilities that reflect the personality of the person being modeled^4–6^. Recent studies have developed role-playing agents based on fictional characters from anime and movies, as well as real or historical figure^7–10^. ”While these systems primarily rely on large language models (LLMs) for text-based chat, avatars operating in the real world require sensing technologies to recognize their surroundings and engage in spoken dialogue that aligns with the characteristics of the person being modeled.

In the field of psychology, which studies individuality, various concepts exist to describe human traits^11^. Personality is defined as “the enduring configuration of characteristics and behavior that comprises an individual’s unique adjustment to life, including major traits, interests, drives, values, self-concept, abilities, and emotional patterns”^12^. Character refers to “the totality of an individual’s attributes and personality traits, particularly their characteristic moral, social, and religious attitudes”^13^, while temperament is defined as “the basic foundation of personality, usually assumed to be biologically determined and present early in life, including such characteristics as energy level, emotional responsiveness, demeanor, mood, response tempo, behavioral inhibition, and willingness to explore”^14^. Individuality is described as “the uniqueness of each individual’s personality”^15^.

The concept of individuality has been particularly emphasized in the fields of caregiving and nursing^16–18^, yet it lacks a clear definition. Kuroda et al. conducted a conceptual analysis to define how individuality is applied in nursing^19^. Their study suggests that analyzing and comparing individuality across different fields can lead to a more refined understanding of the concept, and defined individuality as “the distinct individuality that sets them apart from others, a consistent and authentic representation of the individual, as recognized by others.” Importantly, this definition explicitly includes the perspective of observers, emphasizing that individuality is established through perception and social interaction. Related discussions in social psychology and computer-mediated communication further suggest that individuality and personhood are solely determined by intrinsic traits but are perceived through the coherence and consistency of observable expressions. Goffman’s theory of self-presentation conceptualizes social interaction as a performance through which individuals convey impressions to others, implying that being recognized as a particular person depends on how coherently expressions are enacted and interpreted^20^. Furthermore, research on virtual characters has demonstrated that alignment between observers’ expectations and presented cues plays a central role in judgments of authenticity and person-likeness^21^. Based on these considerations, this study adopts a relational and perceptual definition of individuality, referring to it as a consistent and authentic representation of an individual as recognized by others through interaction. This definition allows us to examine individuality in avatar-based spoken interaction, where observers evaluate whether an avatar’s utterances are perceived as those of the modeled individual under limited and mediated conditions.

In this study, we developed a speech generation system for avatars aimed at real-world human interaction. Through the evaluation of the developed system, we examined the factors that contribute to individuality.

## Related work

In this section, we introduce research related to individuality and role-playing agents, and describe the position of the present work.

### Individuality

Personality research aims to clarify consistent patterns of human behavior, thought, and emotion, and various studies have been conducted in the field of psychology. Allport defined personality as a dynamic organization within the individual’s psychological system that emphasizes individual uniqueness^22^. On the other hand, Cattell attempted to scientifically measure personality traits using statistical methods and proposed 16 fundamental personality factors^23^.

Subsequently, personality research has seen a debate between trait theory and situational theory. Personality is generally considered to exhibit stable characteristics over time, which is broadly agreed upon among researchers^24–26^. According to Costa and McCrae, even when individuals subjectively perceive changes in their identity, trait evaluations show little change^27^. Additionally, personality is considered to demonstrate consistency across various social situations, known as cross-situational consistency^28^. However, Mischel argued that behavior depends more on situational factors than on consistent traits, challenging trait theory^29^. In contrast, Fleeson proposed an interaction model between traits and situations, showing that while traits form the basis of behavior, situations influence their expression^30^. Such approaches have led to a more harmonious integration of trait and situational theories.

In contemporary personality research, the Big Five model has become a widely adopted framework. This model explains personality through five dimensions: extraversion, neuroticism, openness, conscientiousness, and agreeableness^31^, and its validity has been demonstrated across different cultures and languages^32,33^.

The concept of individuality has been discussed across multiple discipline, including nursing science, social psychology, and Human-Computer Interaction (HCI). In nursing research, Kuroda et al. analyzed how the concept of individuality is used in the nursing filed using Roger’s concept analysis method^34^. They defined individuality in nursing as “an internalized core attribute of an individual that possesses uniqueness and consistency, representing the individual’s true self, the image recognized by others, and a state where human dignity is maintained.” While this framework originates in a clinical context, its focus on relationally perceived personhood provides a useful basis for examining how individuals are recognized and distinguished by others in mediated interactions. In social psychology and sociology, individuality and personhood have likewise been conceptualized as perceptual and relational phenomena. Goffman, in his theory of self-presentation, described social interaction as a form of performance in which individuals convey impressions through expressive behaviors, and from this perspective, “individuality” is not determined solely by internal traits but by how coherently self-presentations are enacted and interpreted by observers^20^. This self-presentation framework has been extended to digital media and computer-mediated communication (CMC). Tang et al. showed that the consistency of presented information significantly influences perceived authenticity and liking through experiments using online profiles and short conversational exchanges. In addition, Huang et al. proposed a model of perceived authenticity for virtual characters, demonstrating that the alignment between observers’ expectations and the cues presented by an agent plays a central role in judgments of authenticity and perceived “person-likeness^21^.” Research on authenticity further suggests that judgments of whether an entity is perceived as “authentic” are not determined solely by objective identity but by the degree to which expressions align with an observer’s internal representation. Grayson et al. showed, in the context of consumer research, that authenticity judgments are shaped by symbolic and indexical cues rather than by origin alone^35^. Similarly, Molleda reviewed communication research and characterized authenticity as a multidimensional, perceptual construct constituted through consistency and narrative coherence^36^. Together, these studies suggest that even artificial agents may evoke a strong sense of individuality or personhood when their expressions are consistent with observers’ expectations of a specific individual.

### Role-playing agent

Role-playing agents aim to mimic the speech of specific characters or individuals through the use of LLMs.

Li et al. developed ChatHaruhi, a chatbot that impersonates characters from anime and movies^7^. This study utilized Retrieval Augmented Generation (RAG), leveraging past dialogue from iconic scenes to capture the character’s personality and speech patterns. RAG is a technique that enhances LLM text generation by retrieving relevant information from external databases^37^. Similarly, in the development of RoleLLM by Wang et al., character catchphrases were generated using GPT-4^38^ and integrated with RAG to achieve role-playing capabilities^9^.

Shao et al. developed Character-LLM by creating conversation datasets with LLM and fine-tuning them to portray historical figures such as Beethoven and Cleopatra^8^. Zhou et al. similarly fine-tuned an LLM to create Character-GLM^10^.

Research has also investigated whether LLMs can generate expert-level philosophical texts using a digital corpus of real philosophers. Schwitzgebel et al. fine-tuned GPT-3 Davinci using a digital corpus of works by philosopher Daniel Dennett (15 books and 269 articles) to create a model called DigiDan^39^. The study mentioned that while DigiDan could generate philosophical texts, it had not yet reached a level indistinguishable from a human.

While the above studies enable interaction with agents through text-based chat, avatars acting in the real world require interaction using real-world information, such as visual and auditory information. This research aims to develop avatars that can process information multimodally and interact with humans, based on the assumption that they will operate in the real world.

### Originality of our study

This study investigates whether speech generated by an avatar system designed to model a real individual is perceived as individuality of that individual when compared with the person’s own speech. By grounding individuality in relational perception and observer-based evaluation, this work contributes empirical evidence to discussions of “individuality” in human–agent interaction.

## Speech generation system

**Fig. 1:**
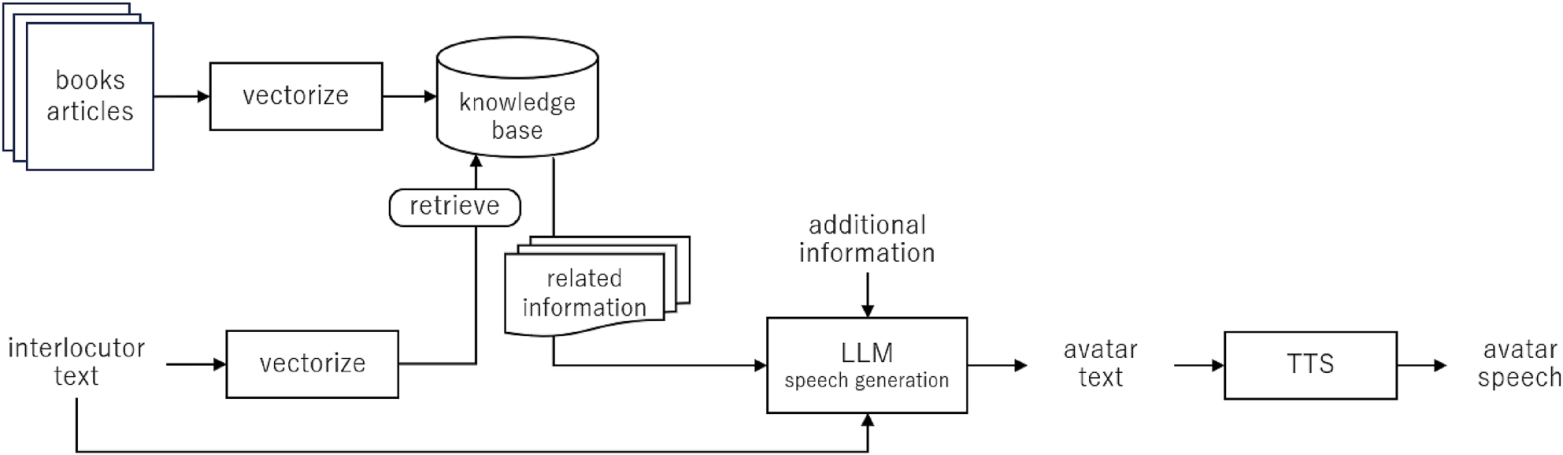
Speech generation system.

In this study, we developed a speech generation system for an avatar modeled after Professor Hiroshi Ishiguro of Osaka University (hereinafter referred to as “the subject”), who has a sufficient corpus available from books and web pages. The developed system is shown in Fig. 1.

It has been reported that Retrieval-Augmented Generation (RAG) consistently outperforms fine-tuning when injecting new knowledge into LLM^40^. In this system, RAG was used to embed the subject’s knowledge and past events into the LLM’s prompts, achieving speech text generation that reflects individuality.

As a preliminary step for speech generation, a knowledge base was created using VectorStoreIndex of LlamaIndex^41^, a data framework for building LLM applications. We created a database using approximately 1.5 million characters worth of data from the subject’s books and online articles. These corpora were segmented into chunks of no more than 1024 tokens (chunk_overlap = 20) and stored in the knowledge base along with their embeddings. The embeddings were obtained using OpenAI’s text-embedding-ada-002 model^42^.

In actual speech generation, the speech text of the interlocutor is used to retrieve highly similar information from the knowledge base, which is then input into the LLM’s prompt. In this study, the conversation partner’s speech was obtained via text input. The text-embedding-ada-002 model was used to obtain the embedding of the partner’s speech text, and cosine similarity was used to calculate relevance.

The LLM used for speech generation is OpenAI’s GPT-4o. In addition to information retrieved from the knowledge base, other essential information for real-world conversation, such as conversation history, visual information from a webcam, the current time, and the elapsed time since the last utterance, was also provided as input (labeled as “additional information” in Fig. 1). However, in this study, a webcam was not used, and only the current time was provided, as the conversation was limited to a single turn (i.e., the interlocutor’s input and the system’s response). Additionally, the subject’s personality and speech patterns were described in the prompt.

The speech generated by AvatarLLM was converted into the subject’s voice using text-to-speech (TTS) technology. AITalk^43^ was used for TTS.

The prompts are shown below. Prompts are instructions given to an LLM, and in OpenAI’s models, these prompts are divided into system, user, and assistant roles. In this study, system and user prompts were created and named “system prompt” and “user prompt,” respectively.

The system prompt contains profile information of the modeled individual, such as name, occupation, and personality, which do not change over time. “State: Sitting” was included as the avatar used is a seated robot (although the experiment described later does not involve a robot). Furthermore, multiple examples of actual utterances were provided to ensure natural speech patterns^44^. To control the LLM’s output, a method of including penalty phrases in the prompt was adopted^45^. In this study, phrases indicating penalties were added to prevent unintended expressions, such as stating that the avatar is an AI.

The user prompt contains directives for generating responses using extracted information from RAG, additional information, and conversation history. The extracted knowledge is inserted into {context_str}, while conversation history is placed into {chat_history}. The placeholder {avatar_name} was replaced with the subject’s name.

In the experiments described later, the GPT baseline model was configured to exclude any information related to the subject, and the system prompt was not used. Additionally, RAG was omitted from the user prompt. Therefore, only the current time was input into {context_str}, and {avatar_name} was set to “A-san.”

**Figure:**
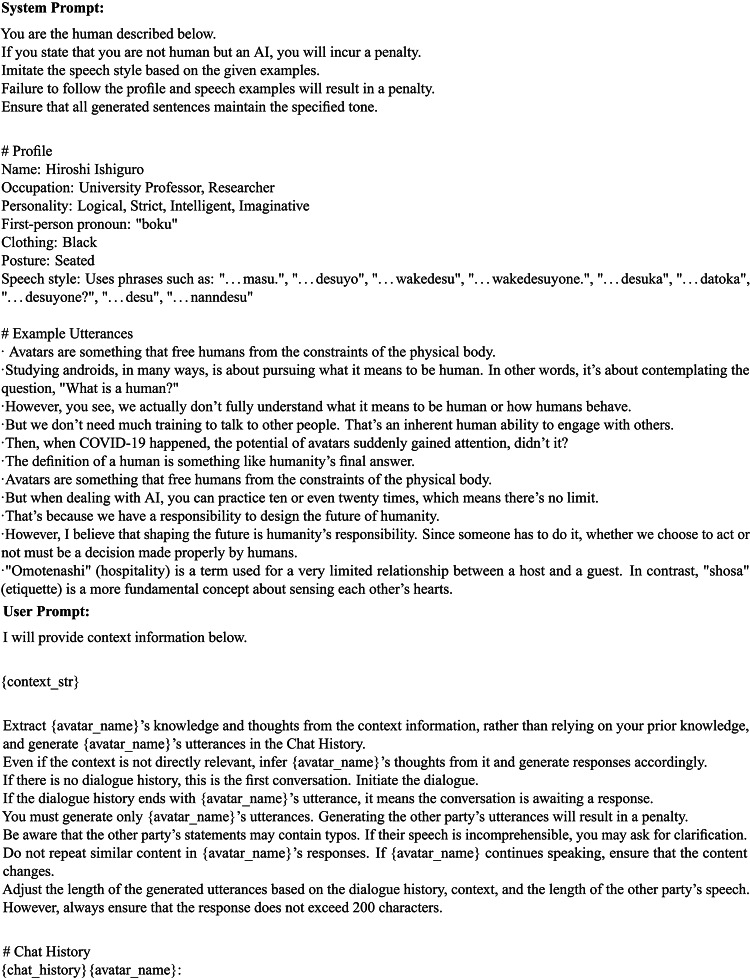

## Experiment

An experiment was conducted to compare the responses of AvatarLLM, GPT-4o (referred to simply as GPT), and the actual person being replicated, in order to investigate the factors underlying individuality. From this point forward, these three entities will be referred to as the speakers. GPT refers to a version of AvatarLLM from which all information specific to the replicated person has been removed (including any knowledge base information from RAG and any personality or speech patterns of the replicated person described in the prompt).

### Method

AvatarLLM was developed with the goal of enabling avatars to interact with humans in the real world. As AvatarLLM is a system designed for voice-based interactions, it outputs spoken language. Therefore, in this experiment, we compared the audio stimulus synthesis of the text generated by each speaker using the TTS of the replicated person. However, for the actual person being replicated, the spoken responses were first transcribed into text and then synthesized into speech. In the user prompt introduced in the previous chapter, “the character count for the text generated by AvatarLLM and GPT was limited to 200 Japanese characters, and the resulting text was synthesized into speech, which lasted about 30 seconds. Accordingly, all audio stimuli were adjusted to a length of approximately 30 seconds. The replicated person was provided with a stopwatch and instructed to give each response in approximately 30 seconds. Additionally, the speech rate and pitch of the synthesized voices were standardized.

Before the evaluation phase, participants were exposed to a short video of the replicated person. This approximately 1-minute video features the person introducing himself and describing his research activities, and is referred to as the pre-training video. The purpose of this phase was to allow participants to form an initial representation of the target individual. In the subsequent evaluation phase, participants evaluated the individuality of audio stimuli generated by the speakers (AvatarLLM, GPT, the replicated person). Importantly, participants did not evaluate the individuality in the pre-training video itself. Instead, they evaluated newly presented audio stimuli after having watched the pre-training video. In this context, recognition refers to “a sense of awareness and familiarity experienced when encountering people, events, or objects that have been encountered before”^46^. The experimental protocol was designed such that participants judged whether the presented audio stimuli were perceived as characteristic of the individual shown in the pre-training video.

In this experiment, four questions listed below were prepared for each speaker. Two of these (S1 and S2) are strongly related to the pre-training video (strong condition), while the other two (W1 and W2) are weakly related (weak condition). Responses to each question were collected from each speaker. Therefore, the experimental conditions involved three speakers and two types of question items, referred to as speaker factors and relevance factors, respectively.

- S1 Please tell me about future direction of your research.
- S2 What kind of robot research and development are you working on?
- W1 Please tell me why you become a researcher.
- W2 Why are you using robots for your research?

Additionally, participants for this experiment were recruited through CrowdWorks^47^. The experiment was conducted using a within-subjects design with Japanese participants aged 20 to 59.

### Procedure

Throughout the experiment, participants were not informed about the identity or the number of speakers from which the audio stimuli were derived. The procedure consisted of a pre-training phase followed by an evaluation phase, as described below.

1. The participants are informed that the study involves evaluating individuality.
2. Participants provide demographic information (age and sex).
3. *Pre-training phase:* Participants watch the pre-training video.
4. After the pre-training phase, participants rate their understanding of the video content on a 7-point scale, ranging from “Not understood at all” to “Completely understood.” Participants who did not sufficiently understand the video content were excluded from the analysis.
5. *Evaluation phase:* Participants are presented with “a question for the speakers” (either S1, S2, W1, or W2), displayed as text on the screen. After reading it, participants listen to the corresponding audio response produced by one of the speakers. During this phase, only the audio response is presented, and the response text is not displayed. Participants then evaluate the audio stimulus. This procedure is repeated for all combinations of four questions (S1 W2) and three speakers, resulting in a total of 12 trials.

All participants provided informed consent prior to the commencement of the study, which was approved by the Ethics Committee of Osaka University, Japan. All methods were carried out in accordance with relevant guidelines and regulations.

### Evaluation method

The individuality for each condition was assessed based on the following five questionnaire items. Q1 and Q2 assess the individuality of the voice, Q3 and Q4 assess the individuality of the content of the speech, and Q5 evaluates whether the overall audio stimulus is perceived as that of the replicated person. All items were rated using a 7-point Likert scale, ranging from 1 (Strongly Disagree) to 7 (Strongly Agree). For Q1 and Q2, as well as Q3 and Q4, the scores of Q2 and Q4 were reverse-coded, and the average of each item pair was used for analysis.

Additionally, following the Directed Questions Scale (DQS) method^48^, attention-check items containing explicit instructions (e.g., “Please choose ‘Agree’ ”) were embedded in the questionnaire, with a total of four such items. Responses that did not correctly follow these instructions were excluded from the analysis.

- Q1 The voice is the subject’s own.
- Q2 The voice is produced by a machine.
- Q3 The content of the speech is the subject’s own.
- Q4 The content of the speech is generated by AI.
- Q5 It is Ishiguro himself.

### Result

The survey results were analyzed for 192 participants (average age: 40.1 years, standard deviation: 8.7 years, 117 men, 72 women, 3 who did not wish to answer), excluding those who answered “Not understood at all” for the pre-training video content, invalid responses, or incomplete answers. A two-way analysis of variance (ANOVA) was conducted for the speaker factors and relevance factors for each of the voice, speech content, and overall evaluations, and significant main effects and interactions were detected for all evaluation items (Table 1).

**Fig. 2:**
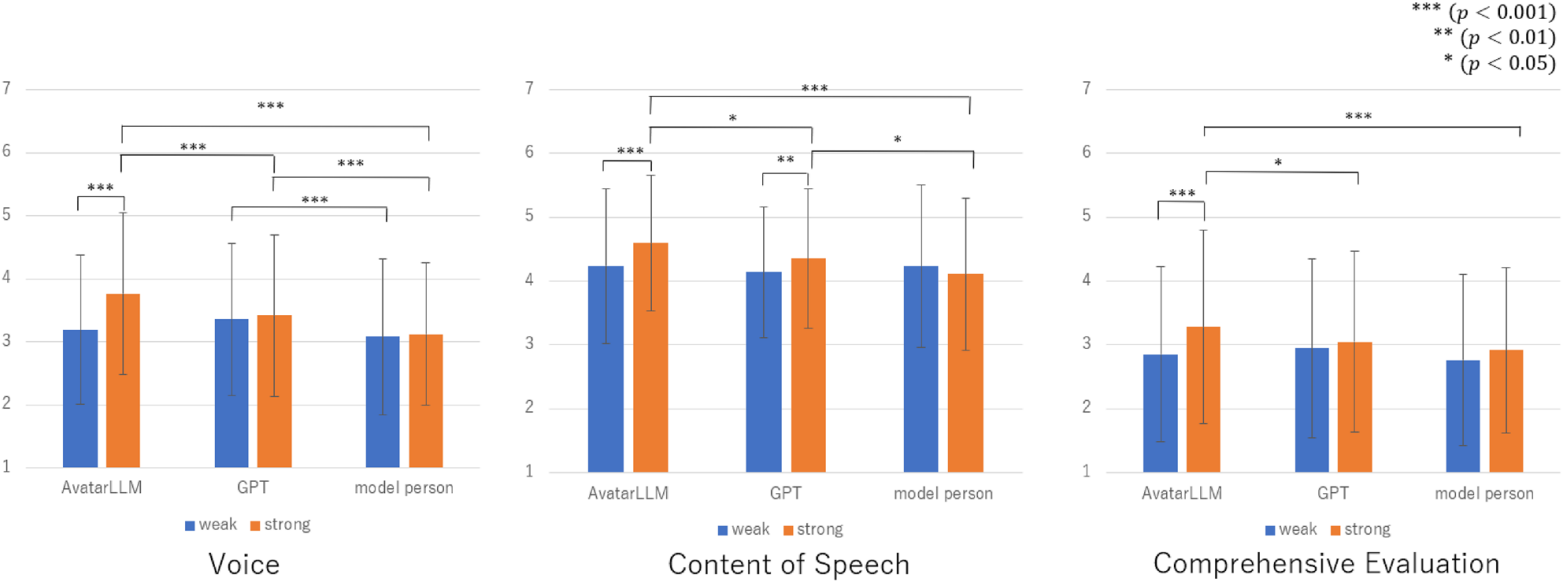
Means, standard deviations, and significant differences for each item of individuality.

Next, multiple comparisons were performed using the Bonferroni correction. The mean values, standard deviations, and significant differences for each item are shown in Fig. 2.

For the voice evaluation, significant differences were found in all combinations of AvatarLLM, GPT, and the replicated person in the strong condition. In the weak condition, significant differences were observed between GPT and the replicated person. Additionally, significant differences were found between the weak and strong conditions for AvatarLLM. For the speech content evaluation, significant differences were observed in all combinations of AvatarLLM, GPT, and the replicated person in the strong condition. In the weak condition, significant differences were found between AvatarLLM and GPT, and between GPT and the replicated person. Additionally, significant differences were found between the weak and strong conditions for both AvatarLLM and GPT. For the overall evaluation, in the strong condition, significant differences were found between AvatarLLM and GPT, and between AvatarLLM and the replicated person. Furthermore, significant differences were observed between the weak and strong conditions for AvatarLLM.

To summarize, in response to questions strongly related to the pre-training video content, the answers from AvatarLLM, GPT, and the replicated person can be ranked by the degree of individuality of their speech content. For both AvatarLLM and GPT, speech content was considered more personally authentic in the strong condition. Similarly, for the voice, the order of individuality was AvatarLLM, GPT, and the replicated person.

**Table 1:** Main and interaction effects of strength and weakness in question conditions and speaker condition.

	Main effect of question	Main effect of speaker	Interaction effects
Voice	*F*(1, 191) = 14.5, *p* < 0.001	*F*(2, 382) = 28.7, *p* < 0.001	*F*(2, 382) = 17.6, *p* < 0.001
Content of speech	*F*(1, 191) = 6.48, *p* < 0.05	*F*(2, 382) = 6.71, *p* < 0.01	*F*(2, 382) = 10.6, *p* < 0.001
Comprehensive evaluation	*F*(1, 191) = 10.4, *p* < 0.01	*F*(2, 382) = 8.44, *p* < 0.001	*F*(2, 382) = 3.92, *p* < 0.01

## Discussion

Regarding speech content, no significant differences were observed among the three speakers for responses weakly related to the pre-training video. However, for both AvatarLLM and GPT, responses strongly related to the pre-training video were perceived as having a higher degree of individuality than those weakly related. In other words, when the speech content had low relevance to the pre-training video, participants might be unable to judge individuality. Conversely, when the content was strongly related, they might be able to evaluate individuality based on the pre-training video. The same trend was observed in the questionnaire item “It is Ishiguro himself.”

Regarding speech content for questions strongly related to the pre-training video, AvatarLLM was perceived as having a higher degree of individuality than the actual person. Similarly, in terms of voice, AvatarLLM was perceived as having a higher degree of individuality than the actual person. One possible reason for this result is that, while the speech of the replicated person changes daily, AvatarLLM generates speech based on a static knowledge base, making its responses more consistent. GPT was also perceived as having a higher degree of individuality than the replicated person. One possible interpretation is that GPT-generated content may have matched a generalized stereotype of a university professor, which could have increased perceived individuality, although further investigation is required.

Additionally, for the voices in response to the strongly related questions, AvatarLLM was perceived as having a higher degree of individuality than the replicated person. GPT was also rated higher than the replicated person. Furthermore, AvatarLLM was rated higher than GPT. As mentioned in the previous chapter, the same TTS system was used for all speakers to synthesize voices based on the replicated person’s voice. Despite this, significant differences in the perception of individuality in voice were observed. This suggests that the individuality of speech content may influence the perception of individuality in voice.

In this experiment, individuality was evaluated based solely on voice. However, for avatars acting in the real world, appearance (i.e., physical embodiment) must also be considered in assessing individuality. The experimental results suggest that even when using the same avatar, the speech content may influence the perceived individuality of its appearance. Furthermore, rather than being limited to one-way speech responses to questions, it is necessary to examine whether individuality can be perceived through interactive dialogue. For instance, one possible evaluation approach is to assess the extent to which an avatar equipped with the developed speech generation system embodies the individuality of the replicated person in conversation.

Additionally, in this experiment, participants evaluated individuality by comparing the audio stimuli with the mental representation formed from the pre-training video, without considering how well they knew the replicated person. If the experiment were conducted with individuals who were well acquainted with the replicated person, the evaluation of the replicated person’s individuality would likely improve, while the evaluation of GPT would decrease. In that case, significant differences in individuality ratings between AvatarLLM and the replicated person would likely disappear.

This study has several limitations. First, individuality was evaluated solely through audio stimuli, without interactive conversation or physical embodiment. Second, participants formed their mental representation of the replicated person’s individuality only from 1-minute video, which may have caused a limited or biased internal model of the person. Third, the experiment involved a single target individual, and therefore the generalization of our findings to other individuals remains uncertain. Future studies should examine multimodal interaction, familiarity level, and multiple target individuals to better understand individuality perception in real-world avatar communication.

## Conclusion

In this study, we evaluated a speech generation system developed for an avatar whose appearance closely resembled that of a real individual, and we examined the factors contributing to “individuality.” The model individual was Professor Hiroshi Ishiguro of Osaka University, and the developed speech generation system was named AvatarLLM.

The experimental results showed that, in terms of speech content, AvatarLLM was perceived as having a higher degree of individuality than the actual replicated person. Similarly, in terms of voice, AvatarLLM was perceived as having a higher degree of individuality than the actual replicated person. These results suggest that consistency in speech is crucial for individuality and that speech content may influence the perception of individuality in voice.

In the future, it will be necessary to examine the individuality of avatars acting in the real world while also considering physical embodiment.

## Data Availability

Data will be made available on reasonable request.

## References

[1] Kumazaki, H. et al. Enhancing communication skills of individuals with autism spectrum disorders while maintaining social distancing using two tele-operated robots. *Front. Psychiatr.* **11**, 598688 (2021). 10.3389/fpsyt.2020.598688 PMC7868394 33569014

[2] Baba, J., Song, S., Nakanishi, J., Yoshikawa, Y. & Ishiguro, H. Local vs. avatar robot: Performance and perceived workload of service encounters in public space. *Front. Robot. AI* **8**, 778753 (2021). 34926593 10.3389/frobt.2021.778753 PMC8678513

[3] Hashimoto, T., Kato, N. & Kobayashi, H. Development of educational system with the android robot Saya and evaluation. *Int. J. Adv. Robot. Syst.* **8**, 28 (2011).

[4] Becker-Asano, C. & Ishiguro, H. Intercultural differences in decoding facial expressions of the android robot Geminoid f. *J. Artif. Intell. Soft Comput. Res.* **1**, 215–231 (2011).

[5] Nishio, S., Ishiguro, H. & Hagita, N. Geminoid: Teleoperated android of an existing person. *Humanoid Robots: New Dev.* **14**, 10–1109 (2007).

[6] Hoeck, K. Android existence: The affect of artificial vitality. HAU. *J. Ethnogr. Theory* **14**, 733–747 (2024).

[7] Li, C. *et al.* Chatharuhi: Reviving anime character in reality via large language model. arXiv:2308.09597 (2023).

[8] Shao, Y., Li, L., Dai, J. & Qiu, X. Character-LLM: A trainable agent for role-playing. arXiv:2310.10158 (2023).

[9] Wang, Z. M. *et al.* Rolellm: Benchmarking, eliciting, and enhancing role-playing abilities of large language models. arXiv:2310.00746 (2024).

[10] Zhou, J. *et al.* Characterglm: Customizing Chinese conversational ai characters with large language models. arXiv:2311.16832 (2023).

[11] American Psychological Association. *Psychology*. https://dictionary.apa.org/psychology (2025). Accessed: 11 Aug 2025.

[12] American Psychological Association. *Personality*. https://dictionary.apa.org/personality (2025). Accessed: 11 Aug 2025.

[13] American Psychological Association. *Character.* https://dictionary.apa.org/character (2025). Accessed: 11 Aug 2025.

[14] American Psychological Association. *Temperament*. https://dictionary.apa.org/temperament (2025). Accessed: 11 Aug 2025.

[15] American Psychological Association. *Individuality*. https://dictionary.apa.org/individuality (2025). Accessed: 11 Aug 2025.

[16] Nakagawa, T., Fujita, A. & Nishizawa, Y. care that respects individuality provided to elderly people with dementia as perceived by Japanese dementia Carers qualified. *Open J. Nurs.* **7**, 1227–1245 (2017).

[17] Uchida, E., Kato, M. & Hara, S. Nursing practices that support the personality of end-stage elderly cancer patients for end-stage. *J. Jpn. Acad. Gerontol. Nur.* **27**, 80–87. 10.20696/jagn.27.1_80 (2022).

[18] Sofronas, M., Wright, D. K. & Carnevale, F. A. Personhood: An evolutionary concept analysis for nursing ethics, theory, practice, and research. *Nurs. Forum* **53**, 406–415 (2018). 29949200 10.1111/nuf.12267

[19] Kuroda, S., Funahashi, M. & Nakagaki, K. Concept analysis of personality in nursing: Using Rodgers’ model. *J. Jpn. Soc. Nurs. Res.* **40**, 107–116 (2017) (**(in Japanese)**).

[20] Goffman, E. The presentation of self in everyday life. In *Social theory re-wired*, 450–459 (Routledge, 2023).

[21] Huang, J. & Jung, Y. Perceived authenticity of virtual characters makes the difference. *Front. Virt. Real.* **3**, 1033709 (2022).

[22] Allport, G. W. *Personality: A psychological interpretation* (Holt, 1937).

[23] Cattell, R. B. *Description and measurement of personality* (World Book Company, 1946).

[24] Conley, J. J. Longitudinal consistency of adult personality: Self-reported psychological characteristics across 45 years. *J. Pers. Soc. Psychol.* **47**, 1325 (1984). 6527217 10.1037//0022-3514.47.6.1325

[25] Graham, E. K. & Lachman, M. E. Personality stability is associated with better cognitive performance in adulthood: Are the stable more able?. *J. Gerontol. Ser. B: Psychol. Sci. Soc. Sci.* **67**, 545–554 (2012). 22357641 10.1093/geronb/gbr149 PMC3441188

[26] Specht, J., Luhmann, M. & Geiser, C. On the consistency of personality types across adulthood: Latent profile analyses in two large-scale panel studies. *J. Pers. Soc. Psychol.* **107**, 540 (2014). 25133730 10.1037/a0036863

[27] Costa, J., Paul T. & McCrae, R. R. *Personality continuity and the changes of adult life* (American Psychological Association, 1989).

[28] Church, A. T. et al. Prediction and cross-situational consistency of daily behavior across cultures: Testing trait and cultural psychology perspectives. *J. Res. Pers.* **42**, 1199–1215 (2008). 22146866 10.1016/j.jrp.2008.03.007 PMC2754878

[29] Mischel, W. *Personality and assessment* (Psychology Press, 2013).

[30] Fleeson, W. Toward a structure-and process-integrated view of personality: Traits as density distributions of states. *J. Pers. Soc. Psychol.* **80**, 1011 (2001). 11414368

[31] Costa, P., McCrae, R. & Psychological Assessment Resources, I. *Revised NEO personality inventory (NEO PI-R) and NEO five-factor inventory (NEO-FFI)* (Psychological Assessment Resources, 1992).

[32] McCrae, R. R. & Allik, I. *The five-factor model of personality across cultures* (Springer Science & Business Media, 2002).

[33] McCrae, R. R. & Terracciano, A. Universal features of personality traits from the observer’s perspective: Data from 50 cultures. *J. Pers. Soc. Psychol.* **88**, 547 (2005). 15740445 10.1037/0022-3514.88.3.547

[34] Rodgers, B. L. & Knafl, K. A. *Concept development in nursing: Foundations, techniques, and applications* (1993).

[35] Grayson, K. & Martinec, R. Consumer perceptions of iconicity and indexicality and their influence on assessments of authentic market offerings. *J. Consum. Res.* **31**, 296–312 (2004).

[36] Molleda, J.-C. Authenticity and the construct’s dimensions in public relations and communication research. *J. Commun. Manag.* **14**, 223–236 (2010).

[37] Gao, Y. *et al.* Retrieval-augmented generation for large language models: A survey. arXiv:2312.10997 (2024).

[38] OpenAI et al. Gpt-4 technical report. arXiv:2303.08774 (2024).

[39] Schwitzgebel, E., Schwitzgebel, D. & Strasser, A. Creating a large language model of a philosopher. *Mind & Lang.* **39**, 237–259 (2024).

[40] Ovadia, O., Brief, M., Mishaeli, M. & Elisha, O. Fine-tuning or retrieval? Comparing knowledge injection in LLMs. arXiv:2312.05934 (2024).

[41] LlamaIndex - Redefine Document Workflows with AI Agents. https://www.llamaindex.ai/. Accessed: 15 Dec 2025.

[42] OpenAI. New and improved embedding model. https://openai.com/index/new-and-improved-embedding-model/ (2022). Accessed: 21 Dec 2024.

[43] Top | AI, Inc. https://www.ai-j.jp/english/. Accessed: 10 Dec 2024.

[44] Brown, T. B. et al. Language models are few-shot learners. arXiv:2005.14165 (2020).

[45] Bsharat, S. M., Myrzakhan, A. & Shen, Z. Principled instructions are all you need for questioning llama-1/2, gpt-3.5/4. arXiv:2312.16171 (2024).

[46] American Psychological Association. *Recognition*. https://dictionary.apa.org/recognition (2025). Accessed: 11 Aug 2025.

[47] CrowdWorks. https://crowdworks.jp/. Accessed: 10 Dec 2024.

[48] Maniaci, M. R. & Rogge, R. D. Caring about carelessness: Participant inattention and its effects on research. *J. Res. Pers.* **48**, 61–83 (2014).

